# Percutaneous Closure of a Large-Bore Carotid Arteriotomy Using a Collagen-Based Vascular Plug

**DOI:** 10.3390/life16020292

**Published:** 2026-02-09

**Authors:** Radoslaw Parma, Radoslaw Gocol, Joanna Nawara-Skipirzepa, Ryszard Bachowski, Wojciech Wojakowski, Damian Hudziak

**Affiliations:** 1 Department of Cardiology and Structural Heart Diseases, Medical University of Silesia, Ziolowa 47, 40-635 Katowice, Poland; joannanawaraa@gmail.com (J.N.-S.);; 2 Department of Cardiac Surgery, Medical University of Silesia, Ziolowa 47, 40-635 Katowice, Poland; gocot@poczta.onet.pl (R.G.);

**Keywords:** carotid artery, vascular closure device, MANTA, iatrogenic injury, central venous catheter, large-bore arteriotomy, aortic dissection, percutaneous closure

## Abstract

**Background:** Inadvertent arterial cannulation during central venous catheter placement is a recognized complication with potentially serious consequences, particularly when involving large-caliber catheters. While management strategies have evolved from mandatory surgical repair to various percutaneous approaches, limited data exist regarding collagen-based vascular closure devices for large-bore carotid arteriotomies. **Case Presentation:** We report the case of a 59-year-old male patient with acute Stanford Type A aortic dissection who underwent emergency surgical repair of the ascending aorta. During central venous cannulation, a five-lumen Certofix Quinto catheter (12-French outer diameter) was inadvertently inserted into the left common carotid artery. Given the complexity of concurrent cardiac surgery and the large-bore nature of the arteriotomy, percutaneous closure with an 18-French MANTA vascular closure device was successfully performed following completion of the aortic repair. The procedure achieved immediate hemostasis without complications. **Outcomes:** The patient remained neurologically intact throughout a 12-month follow-up period. Serial duplex ultrasonography and computed tomography angiography confirmed carotid artery patency without evidence of stenosis, dissection, pseudoaneurysm formation, or thromboembolic complications. **Conclusions:** This case demonstrates the technical feasibility of using a collagen-based vascular closure device for percutaneous management of a large-bore carotid arteriotomy in the acute surgical setting. While the outcome was favorable in this patient, this approach represents an off-label application that requires further validation and should be reserved for carefully selected cases in experienced centers where the benefits of percutaneous closure are judged to outweigh the uncertainties of supra-aortic device deployment.

## 1. Introduction

Central venous catheterization is among the most frequently performed invasive procedures in hospitalized patients, with an estimated annual frequency exceeding 5 million insertions in the United States alone [1]. Despite its ubiquity and the widespread adoption of ultrasound guidance, mechanical complications remain a significant concern, occurring in 5–19% of procedures depending on operator experience and patient anatomy [2]. Inadvertent arterial puncture is the most common mechanical complication, occurring in 3.1–9.4% of internal jugular vein catheterization attempts and 3.1–4.9% of subclavian vein approaches, even with real-time ultrasound guidance [3,4].

While arterial puncture with a small-gauge needle typically carries minimal risk and can be managed with local compression, inadvertent placement of large-caliber catheters into the carotid, subclavian, or brachiocephalic arteries presents a significantly more challenging clinical scenario [5]. The management of such injuries is complicated by the noncompressible anatomic location at the base of the neck, the risk of hemorrhage into the mediastinum or pleural space, and the potential for thromboembolic complications, including stroke [6]. Historically, surgical exploration and direct repair were considered mandatory for large-bore arterial injuries in this location; however, advances in endovascular techniques have expanded the available treatment options [7].

Percutaneous vascular closure devices (VCDs) were initially developed for femoral artery hemostasis following cardiac catheterization procedures. These devices have progressively evolved to accommodate larger arteriotomies, driven by the growth of transcatheter structural heart interventions, particularly transcatheter aortic valve implantation (TAVI) [8]. Two principal categories of VCDs are currently employed for large-bore closure: suture-mediated devices (such as the Perclose ProGlide system, Abbott Vascular, Santa Clara, CA, USA) and collagen-based passive closure devices (such as the MANTA device, Teleflex, Wayne, PA, USA) [9]. Notably, clinical evidence supporting these devices derives almost exclusively from femoral arterial access; data regarding supra-aortic applications remain limited to case reports and small series.

The MANTA vascular closure device is specifically engineered for large-bore arteriotomy closure and represents the first commercially available biomechanical VCD designed for this purpose [10]. The device employs a unique closure mechanism consisting of an intraluminal resorbable polymer toggle, an extraluminal bovine collagen plug, and a radiopaque stainless-steel anchor that secures the assembly at the arteriotomy site [11]. While initially approved and validated for femoral arterial access closure in TAVI and endovascular aneurysm repair procedures, reports of off-label use in alternative vascular territories, including the axillary, subclavian, and carotid arteries, have emerged [12,13,14].

The carotid artery has gained recognition as a valuable alternative access route for TAVI in patients with hostile iliofemoral anatomy [15]. The transcarotid approach, typically performed via surgical cutdown with direct suture repair, has demonstrated procedural success rates exceeding 95% with low stroke rates in experienced centers [16,17]. However, the application of percutaneous closure devices in the carotid territory remains limited, and data regarding collagen-based VCDs for large-bore carotid arteriotomies are particularly scarce.

We present a case of successful percutaneous closure of a large-bore iatrogenic carotid arteriotomy using the 18-French MANTA vascular closure device during emergency aortic surgery. This report aims to describe the technical approach employed and to review the existing literature regarding management strategies for inadvertent large-caliber arterial catheterization, with particular focus on the emerging role of collagen-based closure devices in the carotid territory. This case report was prepared in accordance with the CARE (CAse REport) guidelines [18]. A completed CARE checklist is provided as Supplementary Material S1.

## 2. Case Presentation

### 2.1. Patient Presentation and Initial Assessment

A 59-year-old male patient presented to the emergency department with an acute onset of severe chest pain radiating to the back, associated with diaphoresis and hypertension (Table 1). His past medical history was significant for longstanding hypertension and hyperlipidemia, managed with amlodipine 10 mg daily and atorvastatin 40 mg daily. He denied any history of coronary artery disease, prior cardiac surgery, or known connective tissue disorders. The patient was a non-smoker with no family history of aortic disease. Given suspected acute aortic dissection, an expedited diagnostic workup protocol was initiated [19].

Emergent computed tomography angiography (CTA) of the chest confirmed an acute Stanford Type A aortic dissection with the intimal flap extending from the aortic root to the proximal descending thoracic aorta. Both carotid arteries were supplied by the true lumen, and there was no evidence of malperfusion syndrome. Transthoracic echocardiography demonstrated moderate aortic regurgitation with preserved left ventricular function.

Following multidisciplinary consultation, the patient was emergently referred for surgical replacement of the ascending aorta. Informed consent was obtained, and the patient was transferred to the operating room within 90 min of diagnosis.

### 2.2. Intraoperative Events and Recognition of Arterial Injury

Under general anesthesia, preparation for cardiopulmonary bypass commenced. During central venous cannulation of the left internal jugular vein, a five-lumen Certofix Quinto catheter (12-French outer diameter; B. Braun Melsungen AG, Melsungen, Germany) was inadvertently inserted. Despite attempted ultrasound guidance, the proximity and overlap of the internal jugular vein and common carotid artery in the patient’s anatomy led to inadvertent arterial access. Initial aspiration yielded dark, non-pulsatile blood, which was interpreted as venous return. The catheter was advanced without resistance.

Central venous access was attempted using a short-axis ultrasound approach, selected due to emergent circumstances and limited patient neck positioning. Initial needle passage appeared to enter the compressible anterior structure; yet, needle tip visualization was suboptimal due to the acute angle of insertion. The aspiration of dark, non-pulsatile blood was attributed to the patient’s relative hypotension and ongoing preparation for hypothermic bypass, leading to misinterpretation as venous return.

Recognition of the arterial malposition occurred during attempted pressure transduction, which revealed an arterial waveform with a mean pressure of 85 mmHg. Subsequent blood gas analysis confirmed arterial placement. Fluoroscopic imaging localized the catheter tip within the aortic arch, with the insertion site in the left common carotid artery approximately 3 cm proximal to the carotid bifurcation.

Given the urgent nature of the aortic pathology and the need to initiate cardiopulmonary bypass, a decision was made to leave the arterial catheter in situ and address the carotid injury following completion of the primary surgical repair. The patient was systemically heparinized (300 units/kg, target ACT > 480 s) for cardiopulmonary bypass. The catheter remained secured in position to prevent uncontrolled hemorrhage and to maintain a potential route for arterial access or for guidance during repair, if needed.

The ascending aorta was successfully replaced with a 28 mm Hemashield Platinum woven polyester graft (Getinge AB, Göteborg, Sweden) under deep hypothermic circulatory arrest (20 °C) with antegrade selective cerebral perfusion. Intraoperative transesophageal echocardiography confirmed competent aortic valve function with no residual aortic regurgitation. The patient was successfully weaned from bypass with stable hemodynamics.

### 2.3. Percutaneous Closure of the Carotid Arteriotomy

Following protamine reversal (target ACT < 140 s) and hemostatic optimization, attention was directed to the carotid arteriotomy. The multidisciplinary team, comprising cardiac surgeons and an interventional cardiologist, deliberated on the optimal management strategy. Surgical cutdown and direct repair were considered but were associated with potential concerns, including neck hematoma formation in an anticoagulated field, proximity to recent surgical sites, and prolongation of an already extensive procedure in a high-risk patient.

After careful evaluation, percutaneous closure using the MANTA vascular closure device was selected. The decision was based on the documented success of collagen-based VCDs in achieving hemostasis of large-bore arteriotomies, the device’s mechanism of action, which does not depend on suture deployment into potentially diseased vessel walls, and the team’s extensive experience with the device in femoral applications for TAVI procedures.

Before the closure, anticoagulation status was verified with an ACT measurement of 150 s, confirming adequate protamine reversal. Ensuring normal mean arterial pressure during device deployment allowed for minimizing the risk of bleeding while maintaining cerebral perfusion.

The procedure was performed in the operating room. Using the dedicated 8-French depth locator, the skin-to-arteriotomy distance was measured at 1 cm. This measurement was essential for appropriate intra-arterial toggle deployment depth and optimal collagen plug positioning at the external vessel wall. Considering a 12-French outer diameter of the indwelling catheter, the 18-French MANTA system was selected to ensure adequate arteriotomy coverage.

The existing arterial catheter was removed over a 0.035-inch J-tipped guidewire, maintaining access. The MANTA delivery sheath was advanced over the wire into the left common carotid artery. The resorbable polymer toggle was deployed at a depth of 2 cm from the skin entry point, ensuring it was securely positioned within the arterial lumen. Controlled traction was then applied to seat the toggle against the internal vessel wall while advancing the collagen plug to approximate the external arterial surface. The stainless-steel anchor was deployed to lock the closure unit in position.

Immediate hemostasis was achieved without the need for manual compression. Technical success was achieved with the absence of any bleeding at the skin entry site and confirmation of laminar flow in the left common carotid artery on immediate duplex ultrasonography. Total time from MANTA sheath insertion to confirmed hemostasis was approximately 4 min.

Embolic prevention measures specific to supra-aortic deployment included: thorough flushing of the delivery sheath with heparinized saline before insertion; aspiration through the sheath to remove any air or debris before device advancement, and minimization of total procedure time to reduce thrombus formation risk on intravascular components.

Neurological monitoring during the closure procedure included continuous pupillary assessment and intraoperative neuromonitoring. Immediately following device deployment, a focused neurological examination by the anesthesiology team confirmed reactive pupils and intact brainstem reflexes. Stroke assessment using the National Institutes of Health Stroke Scale (NIHSS) was performed upon extubation, which was unremarkable (NIHSS score 0).

### 2.4. Postoperative Course and Follow-Up

The patient’s immediate postoperative course was uneventful. He was extubated within 8 h of surgery and transferred to the step-down unit on postoperative day 2. Serial neurological examinations throughout the hospital stay revealed no focal deficits, and the patient remained cognitively intact. No neck hematoma or swelling developed at the arteriotomy site.

Duplex ultrasonography of the carotid arteries was performed on postoperative day 3 and demonstrated normal flow velocities in the left common carotid artery without evidence of stenosis, intimal dissection, or pseudoaneurysm formation. Duplex parameters demonstrated normal peak systolic velocity (PSV) and end-diastolic velocity (EDV) in the left common carotid artery, with no focal velocity elevation suggestive of stenosis (PSV ratio < 1.5 compared to the proximal reference segment). The extravascular collagen plug was visualized as an echogenic structure adjacent to the anterior vessel wall. Computed tomography angiography prior to discharge confirmed patency of the carotid arteries bilaterally, satisfactory positioning of the MANTA device components, and no evidence of vascular complications.

The patient was discharged on postoperative day 9 in stable condition. Prescribed medications at discharge included aspirin 75 mg daily, without specific anticoagulation initiated for the carotid closure site. Aspirin monotherapy was selected based on the mechanical nature of the MANTA closure mechanism, the absence of pre-existing indications for dual antiplatelet therapy, the elevated bleeding risk following recent major cardiac surgery, and the lack of established guidelines for antiplatelet therapy following VCD deployment in the carotid territory.

**Figure 1:**
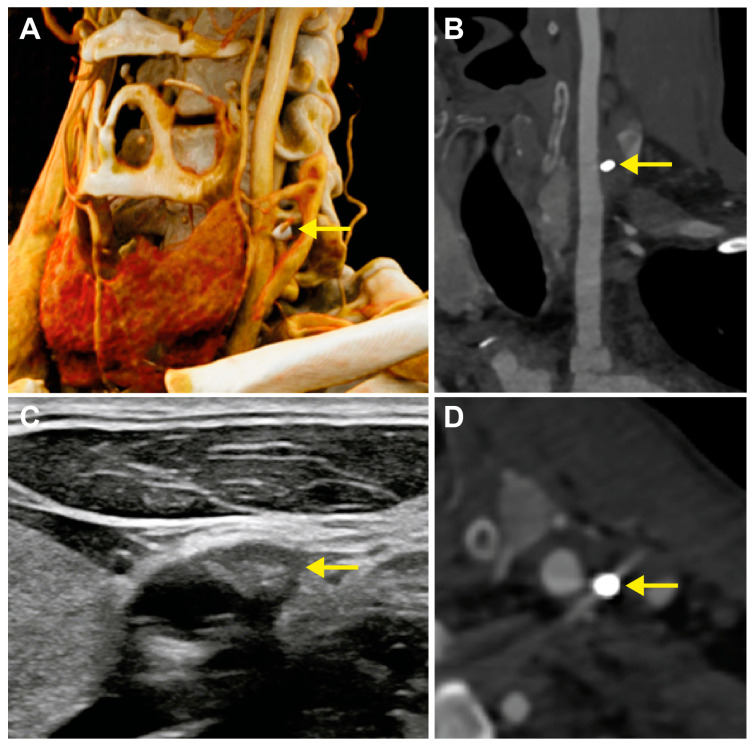
Imaging follow-up. (**A**,**B**,**D**) Computed tomography angiography at 12-months demonstrating the metallic anchor of the MANTA 18-F closure device adjacent to the left common carotid artery (arrows). The carotid artery demonstrates complete patency without stenosis or pseudoaneurysm formation. (**C**) Duplex ultrasonography on postoperative day 3 showing the extravascular collagen plug (arrow) with normal carotid artery flow.

Clinical and imaging follow-up was conducted at 6 and 12 months postoperatively. The patient remained asymptomatic throughout the follow-up period, with no neurological events, transient ischemic attacks, or symptoms of carotid insufficiency. Repeat duplex ultrasonography at 12 months demonstrated continued patency of the left common carotid artery without progressive stenosis. The collagen plug demonstrated resorption compared to early postoperative imaging. At 12-month follow-up, computed tomography angiography (Figure 1A,B,D) confirmed complete patency of the left common carotid artery with no evidence of stenosis greater than 10%, no pseudoaneurysm formation, and no dissection. The radiopaque metallic anchor remained clearly visible adjacent to the vessel wall, with complete resorption of the collagen components.

## 3. Discussion

This case demonstrates the successful use of a collagen-based vascular closure device for percutaneous management of an iatrogenic large-bore carotid arteriotomy during emergency cardiac surgery. To our knowledge, this represents one of the first reported uses of the MANTA device specifically for closure of an inadvertent large-caliber central venous catheter malposition in the carotid artery.

### 3.1. Management of Inadvertent Arterial Catheterization

The management of inadvertent arterial catheterization has evolved considerably over the past two decades. Historically, any arterial placement of a central venous catheter, regardless of size, was managed with surgical exploration and direct repair [20]. This approach, while definitive, is associated with significant morbidity, including wound complications, cranial nerve injury, and prolonged operative time. Moreover, in critically ill patients or those undergoing concurrent major surgery, the added physiological stress of neck exploration may be poorly tolerated.

Contemporary guidelines now stratify management based on catheter size and patient-specific factors [21]. For small-bore catheters (typically <9 French), removal with manual compression or compression-assisted devices may be appropriate in patients without significant coagulopathy or hostile neck anatomy. For larger catheters, the risk of uncontrolled hemorrhage into the mediastinum necessitates either surgical repair or endovascular intervention [22].

Several endovascular approaches have been described for the management of large-bore arterial injuries, including balloon tamponade with or without covered stent placement, suture-mediated closure devices, and collagen-based plug devices [23,24]. Each approach has specific advantages and limitations. Balloon tamponade provides temporary control but does not achieve definitive closure. Covered stents provide reliable hemostasis but may compromise vessel patency in smaller-caliber arteries and introduce long-term concerns regarding stent-related complications. Suture-mediated devices have been successfully employed in the carotid territory but require adequate tissue puncture and may be challenging in diseased or calcified vessels [25].

### 3.2. The MANTA Vascular Closure Device

The MANTA device differs fundamentally from suture-mediated closure systems in its mechanism of hemostasis. Rather than deploying sutures through the vessel wall, the MANTA achieves closure via a mechanical “sandwich” configuration comprising an intraluminal toggle, the arterial wall, and an extraluminal collagen plug, secured by a metallic anchor [26]. This passive approximation mechanism offers several potential advantages in off-label applications: it does not require needle deployment into potentially diseased vessel walls, it provides immediate hemostasis without dependence on coagulation function, and it accommodates a range of arteriotomy sizes (10–25-French outer diameter) [27].

The SAFE MANTA pivotal trial established the safety and efficacy of the device for femoral arteriotomy closure following TAVI and endovascular aneurysm repair [28]. Among 263 patients, time to hemostasis was rapid (median 24 s), and the rate of major access-site complications was 4.2% at 60-day follow-up. Notably, device success was achieved in 97.7% of cases. Subsequent real-world registries have corroborated these findings, with technical success rates exceeding 95% and favorable safety profiles compared to suture-mediated alternatives [29,30]. Importantly, these outcomes derive exclusively from femoral applications; extrapolation to supra-aortic territories should be approached with caution, given distinct anatomic and embolic risk considerations.

Off-label applications of the MANTA device have expanded to include transaxillary and transsubclavian TAVI access, with several case series reporting successful percutaneous closure [12,31]. Most recently, the device has been employed for percutaneous decannulation of venoarterial extracorporeal membrane oxygenation (VA-ECMO), demonstrating reduced vascular complications compared to surgical decannulation in selected patients [32]. These experiences suggest that the MANTA’s closure mechanism may be broadly applicable across diverse vascular territories; still, the cerebrovascular implications of supra-aortic deployment warrant particular attention.

### 3.3. Carotid Artery Access and Closure

The carotid artery has emerged as an increasingly utilized alternative access route for TAVI in patients with prohibitive iliofemoral anatomy [33]. Contemporary data from the French TAVI registry demonstrate that transcarotid procedures account for approximately 3.4% of all TAVI cases, with procedural success rates approaching 97% and 30-day mortality rates comparable to transfemoral approaches [34]. Importantly, stroke rates following transcarotid TAVI have been reassuringly low (1.6–2.0%) in experienced centers, likely reflecting the proximity of the access site to the target anatomy and the reduced catheter manipulation required [16]. However, these data pertain to planned surgical access with direct visualization and suture repair; extrapolation to percutaneous closure following inadvertent cannulation requires caution.

Traditional transcarotid TAVI involves surgical cutdown of the common carotid artery, followed by direct suture repair after sheath removal [35]. While this approach provides reliable hemostasis under direct visualization, it necessitates surgical expertise, typically requires general anesthesia, and may be associated with wound complications and cranial nerve injury. The potential for fully percutaneous transcarotid TAVI, analogous to contemporary transfemoral practice, has prompted interest in carotid artery closure devices [36].

Reports of VCD use in the carotid artery remain limited but are increasing. Suture-mediated closure with ProGlide devices has been described for both elective transcarotid TAVI and management of inadvertent arterial catheterization, with generally favorable outcomes [37,38]. A recent systematic review of closure device use for inadvertent carotid catheterization identified fewer than 20 published cases, predominantly managed with suture-based systems [39]. The current case adds to this nascent literature by demonstrating the successful use of a collagen-based alternative in a large-bore arteriotomy.

### 3.4. Technical Considerations and Procedural Insights

Several technical considerations merit discussion regarding MANTA deployment in the carotid territory. First, accurate measurement of the skin-to-arteriotomy depth is critical for appropriate toggle positioning. Similar to the femoral artery, where access depths are not easily predictable, the depth to the carotid artery varies considerably based on patient body habitus and neck anatomy. In our case, the measured depth of 1 cm necessitated deployment of the toggle at 2 cm from skin entry to ensure adequate intraluminal positioning: the minimum deployment depth recommended for the MANTA system [40].

Second, device sizing must account for the arteriotomy created by the indwelling catheter. The 18-French MANTA system is designed to close arteriotomies created by devices with outer diameters ranging from 14–25 French. While the 12-French outer diameter of the Certofix catheter falls slightly below this range, the arteriotomy tract was likely dilated during initial catheter placement, making the 18-French MANTA an appropriate selection. Additionally, the larger device ensures adequate collagen coverage of the external arteriotomy [41].

Third, the unique hemostatic challenges of the intraoperative setting must be acknowledged. Our patient had received full systemic heparinization for cardiopulmonary bypass, with residual anticoagulation effects despite protamine reversal. The MANTA’s mechanical closure mechanism, supplemented by the procoagulant properties of bovine collagen, appeared effective in this setting. This contrasts with suture-mediated devices, which rely on tissue approximation and may be more susceptible to failure in coagulopathic patients [42].

Patient selection for percutaneous carotid closure requires careful consideration of anatomic contraindications. Relative contraindications for MANTA deployment in this territory include severe circumferential vessel calcification, pre-existing significant carotid stenosis, proximity of the arteriotomy to the carotid bifurcation, and evidence of vessel wall disease, dissection, or thrombus. In our case, preoperative CTA confirmed that both carotid arteries were supplied by the true lumen without significant atherosclerotic disease, and the arteriotomy was located approximately 3 cm proximal to the bifurcation, favoring percutaneous management.

Finally, imaging follow-up is crucial to ensure patient safety. Carotid artery complications may evolve, and early detection of dissection, stenosis, or pseudoaneurysm formation allows timely intervention. Our surveillance protocol, incorporating both duplex ultrasonography and computed tomography angiography, provided a comprehensive evaluation of the closure site during the follow-up period [43].

### 3.5. Limitations and Future Directions

This report describes a single case experience and must be interpreted within the context of its inherent limitations. Case reports provide proof of concept but cannot establish safety or efficacy for broader application. The successful outcome in this patient may not be generalizable to patients with different anatomic or clinical characteristics, including those with significant carotid artery disease, vessel calcification, or ongoing anticoagulation requirements.

Furthermore, the MANTA device is not approved for use in the carotid artery, and off-label applications carry inherent uncertainty regarding device performance in untested vascular beds. Specific concerns in the carotid territory include the potential for embolic complications during deployment, the long-term fate of retained device components in proximity to the cerebral circulation, and the impact of the permanent metallic anchor on future imaging or reintervention [44].

Prospective registries or clinical trials evaluating percutaneous closure devices for carotid artery closure are warranted to establish appropriate patient selection criteria, standardize deployment techniques, and characterize the spectrum of potential complications. Comparative studies examining collagen-based versus suture-mediated closure in this territory would be particularly valuable. Additionally, longer-term follow-up data are needed to assess the durability of closure and to detect any late adverse events.

### 3.6. Risk Mitigation and Benefit-Risk Considerations

The decision to use a collagen-based VCD in the carotid territory requires consideration of embolic and neurological risks related to supra-aortic device deployment. In our case, several factors favored percutaneous closure: the patient had already undergone a prolonged, high-risk cardiac surgical procedure, and preoperative imaging confirmed healthy carotid arteries supplied by the true lumen, with no significant atherosclerotic burden. Also, the arteriotomy was in a favorable position with adequate working distance, and our team already had extensive experience with the MANTA device in femoral applications. We aimed to minimize procedural risks by thorough sheath flushing, aspiration before device advancement, and shortening the time of intravascular device positioning. The MANTA VCD radiopaque stainless-steel pin remains permanently at the closure site without complicating future cervical imaging or reintervention, as evidenced by studies in transfemoral access TAVI cohorts. In our patient, the device has not caused clinical issues through 12 months of follow-up, but longer surveillance is warranted. The polymer toggle and collagen plug are designed to resorb over time, and imaging at 12 months confirmed collagen resorption.

## 4. Conclusions

This case report describes the technically successful percutaneous closure of a large-bore iatrogenic carotid arteriotomy using the MANTA vascular closure device during emergency cardiac surgery for acute aortic dissection. The procedure achieved immediate hemostasis, and a 12-month follow-up confirmed carotid artery patency without stenosis, dissection, or neurological sequelae. While this favorable outcome is encouraging, it represents a single case of off-label device use, and the findings cannot be generalized without further validation.

As interventional techniques continue to evolve and transcatheter therapies expand to encompass increasingly complex procedures and alternative access routes, the role of percutaneous closure devices in the supra-aortic circulation merits further investigation. While surgical repair remains the established standard for carotid artery injuries, endovascular alternatives, including collagen-based VCDs, may offer advantages in appropriately selected patients, particularly those with competing surgical priorities or prohibitive surgical risk.

Prospective studies are needed to validate the safety and efficacy of this approach, define optimal patient selection criteria, and compare outcomes across available closure technologies. Until such data become available, the use of the MANTA device in the carotid artery should be reserved for carefully selected cases in which the benefits of percutaneous closure are judged to outweigh the uncertainties of off-label device use.

## Figures and Tables

**Table 1:** Patient and Procedure Timeline.

Time Point	Event/Parameter
T = 0	Emergency department presentation; CTA confirms Type A dissection
T + 90 min	OR transfer; general anesthesia induction
T + 110 min	CVC insertion attempt; inadvertent carotid cannulation
T + 130 min	Heparinization (300 U/kg); ACT > 480 s; CPB initiated
T + 4.5 h	Aortic repair completed; separation from CPB
T + 5 h	Protamine reversal; ACT 127 s confirmed
T + 5.25 h	MANTA 18F deployment; hemostasis in 4 min
POD 0 + 8 h	Extubation; NIHSS 0
POD 3	Duplex: PSV 78 cm/s, EDV 24 cm/s, no stenosis
POD 7	CTA: patent carotid, no complications
POD 9	Hospital discharge; aspirin 75 mg daily
6 months	Follow-up: asymptomatic, duplex unchanged
12 months	CTA: complete patency, collagen resorbed, anchor visible

ACT, activated clotting time; CPB, cardiopulmonary bypass; CTA, computed tomography angiography; CVC, central venous catheter; EDV, end-diastolic velocity; NIHSS, National Institutes of Health Stroke Scale; OR, operating room; POD, postoperative day; PSV, peak systolic velocity.

## Data Availability

Data supporting the findings of this study are not publicly available due to patient privacy and ethical restrictions. All relevant clinical information is included within the article.

## Supplementary Materials

The following supporting information can be downloaded at: https://www.mdpi.com/article/10.3390/life16020292/s1, Supplementary Material S1: Completed CARE checklist.
