# Supplementary material for: Percutaneous Closure of a Large-Bore Carotid Arteriotomy Using a Collagen-Based Vascular Plug
Source: Life (Basel). 2026 Feb 9;16(2):292. doi: 10.3390/life16020292 (PMC12942100; doi:10.3390/life16020292)
Supplement: Supplementary file 1 [file life-16-00292-s001.zip › life-4130581-supplementary.pdf]

Supplementary Material S1

CARE Checklist for Case Reports

Title: Percutaneous Closure of a Large-Bore Carotid Arteriotomy Using a Collagen-Based Vascular Plug

Reference: Gagnier JJ, et al. The CARE guidelines: consensus-based clinical case reporting guideline development. BMJ Case Rep. 2013.

Item	Topic	Checklist Item Description	Location	Reported?
1	Title	The diagnosis or intervention of primary focus followed by 'case report'	Title	Yes
2	Key Words	2 to 5 key words that identify diagnoses or interventions	Keywords	Yes
3a	Abstract - Introduction	What is unique and why is it important?	Abstract	Yes
3b	Abstract - Case	Main symptoms, clinical findings, main diagnoses, interventions, outcomes	Abstract	Yes
3c	Abstract - Conclusion	What is the main take-away lesson(s)?	Abstract	Yes
4	Introduction	Brief summary of background and rationale	Section 1	Yes
5a	Patient Information - Demographics	Anonymized patient demographics	Section 2.1	Yes
5b	Patient Information - Chief Complaints	Main symptoms of the patient	Section 2.1	Yes
5c	Patient Information - Medical History	Medical, family, and psychosocial history	Section 2.1	Yes
5d	Patient Information - Comorbidities	Relevant past interventions and their outcomes	Section 2.1	Yes
6	Clinical Findings	Physical examination and relevant clinical findings	Section 2.1	Yes
7	Timeline	Chronological order of events (Table 1)	Table 1	Yes
8a	Diagnostic Assessment - Methods	Diagnostic methods (CTA, echo, labs)	Section 2.1-2.2	Yes
8b	Diagnostic Assessment - Challenges	Diagnostic challenges (ultrasound-guided access)	Section 2.2	Yes
8c	Diagnostic Assessment - Reasoning	Diagnostic reasoning, including differential	Section 2.2	Yes

8d	Diagnostic Assessment - Prognosis	Prognostic characteristics, where applicable	Section 2.2	Yes
9a	Therapeutic Intervention - Types	Types of intervention (surgical repair, MANTA closure)	Section 2.2-2.3	Yes
9b	Therapeutic Intervention - Administration	Administration of intervention and changes	Section 2.3	Yes
9c	Therapeutic Intervention - Strengths/Limits	Strengths and limitations of the intervention	Section 3.2, 3.4	Yes
10a	Follow-up and Outcomes - Summary	Clinician and patient-assessed outcomes	Section 2.4	Yes
10b	Follow-up and Outcomes - Testing	Important follow-up test results (duplex, CTA)	Section 2.4, Fig 1	Yes
10c	Follow-up and Outcomes - Adherence	Intervention adherence and tolerability	Section 2.4	Yes
10d	Follow-up and Outcomes - Adverse Events	Adverse and unanticipated events	Section 2.4	Yes (none)
11a	Discussion - Strengths	Discussion of strengths of case management	Section 3.4	Yes
11b	Discussion - Limitations	Discussion of limitations of case management	Section 3.5	Yes
11c	Discussion - Literature	Relevant medical literature	Sections 3.1-3.3	Yes
11d	Discussion - Rationale	Scientific rationale for conclusions	Section 3.4, 3.6	Yes
12	Patient Perspective	Patient perspective on treatment (not applicable)	N/A	N/A
13	Informed Consent	Patient consent obtained	End matter	Yes
